# Smoking cessation and lung cancer risk in an Asian population: Findings from the Singapore Chinese Health Study

**DOI:** 10.1038/sj.bjc.6605782

**Published:** 2010-09-14

**Authors:** K-Y Wong, A Seow, W-P Koh, A Shankar, H-P Lee, M C Yu

**Affiliations:** 1 Department of Epidemiology and Public Health, Yong Loo Lin School of Medicine, National University of Singapore, MD3, 16 Medical Drive, Singapore 117597, Singapore; 2 Department of Community Medicine, West Virginia University School of Medicine, Morgantown, West Virginia, WV, USA; 3 The Masonic Cancer Center, University of Minnesota, Minneapolis, MN, USA

**Keywords:** tobacco use, cohort study, smoking, quitting, lung cancer

## Abstract

**Background:**

Smoking cessation is an important strategy for reducing the harmful effects of tobacco, particularly in the prevention of lung cancer; however, prospective data on the impact of smoking cessation on lung cancer risk in Asian populations are limited.

**Methods:**

We studied a population-based cohort of Chinese men and women aged 45–74 years – participants of the Singapore Chinese Health Study. Information on smoking, lifestyle and dietary habits was collected at the time of recruitment in 1993–1998; and smoking status was assessed again at a second interview in 1999–2004 (mean interval 5.8 years). Participants were followed up to 31 December 2007, and incident cases of lung cancer were ascertained by linkage with population-wide registries.

**Results:**

Among 45 900 participants, there were 463 incident cases of lung cancer. Relative to current smokers, those who quit smoking subsequent to baseline assessment had a 28% decrease in the risk of lung cancer (adjusted hazard ratio (HR) 0.72; 95% CI (95% confidence interval): 0.53–0.98). The risk was less than half in ex-smokers who had quit before the first interview and maintained their status (HR 0.42; 95% CI: 0.32–0.56).

**Conclusions:**

Reduction in lung cancer incidence with smoking cessation in Asian populations is substantial and can be observed within a few years after quitting.

The World Health Organisation recognises tobacco use as the major preventable cause of adult death from cancer. Approximately 80% of male and 45% of female lung cancer cases worldwide are attributable to smoking (Boyle and Levin, 2008). Evidence from epidemiological studies indicates that within 10 years of quitting smoking, there is a 40–90% reduction in lung cancer risk, and that the magnitude of risk reduction varies with the intensity of smoking, time since stopping and age at cessation (Sobue *et al*, 2002; Ebbert *et al*, 2003; Wen *et al*, 2005; Tverdal and Bjartveit, 2006; Lam *et al*, 2007; Gotfredsen *et al*, 2008; Kenfield *et al*, 2008; Song *et al*, 2008).

Estimates of the benefits of smoking cessation are expected to be largest in countries where the majority of smokers have been exposed for a long period of time (International Agency for Research on Cancer, 2007). This is the case in Western populations, which are at a more advanced phase of the ‘tobacco epidemic’ (Huxley *et al*, 2007). In Asia, where the maximal impact is yet to be felt, the full magnitude of the effect, and its relation to time since quitting, has yet to be well documented.

In Singapore, lung cancer remains the most frequent cancer among men and the third most frequent among women (Seow *et al*, 2004). Smoking prevalence stood at 21.8 and 3.5%, respectively, in 2004 (Ministry of Health, Department of Epidemiology and Disease Control, 2005); these rates are among the lowest globally (Jha *et al*, 2002; Slama, 2008). Active measures by the government include laws restricting smoking in public places, prohibition of tobacco advertisements and promotion of healthy lifestyles (Bhalla *et al*, 2006). We used data from a prospective population-based cohort of middle-aged to elderly Chinese in Singapore to evaluate the effect of cessation on lung cancer risk, and the potential impact of measures to reduce the harmful effect of tobacco in Asian populations.

## Materials and methods

### Study population

The Singapore Chinese Health Study is a prospective cohort study, and the design and methodology have been described previously (Hankin *et al*, 2001). Briefly, the cohort was drawn from permanent residents or citizens of Singapore who live in government-built housing estates. Recruitment was restricted only to men and women of Chinese ethnicity (from the two major dialect groups, Hokkiens and Cantonese) aged between 45 and 74 years. The population was enrolled between April 1993 and December 1998, and subsequently re-interviewed in 1999–2004.

### Exposure assessment

A face-to-face interview was conducted in the subject's home at the time of recruitment by a trained interviewer using a structured questionnaire, which requested information on demographics, educational attainment, lifetime use of tobacco, current use of alcohol, current level of physical activity, medical history and family history of cancer. The questionnaire included a validated, semi-quantitative food frequency section listing 165 food items commonly consumed in the study population. The second re-interview, which assessed cigarette smoking as well as other selected lifestyle factors and medical histories, was conducted from 1999 to 2004, either in-person or over the telephone, using standardised questionnaire and method.

In both surveys, we obtained information on smoking habits – participants were asked whether they smoked, average number of cigarettes smoked per day, age they started smoking regularly and years since quitting for ex-smokers. Although the style of the questions differed slightly between the two surveys, all these variables were included in both questionnaires.

In the baseline questionnaire, subjects were grouped into three groups according to the answers given to the following question, ‘Have you ever smoked at least 1 cigarette a day for 1 year or longer?’ Subjects who answered ‘no’ were classified as ‘never smokers’, those who answered ‘yes, but I quit smoking’ were classified as ‘former smokers’ and those who answered ‘yes, and I currently smoke’ were classified as ‘current smokers’. At the second assessment, the population was again grouped based on the following two questions, ‘Have you ever smoked more than 100 cigarettes in your lifetime?’ and divided into three groups; those who answered ‘no’ were classified as ‘never smokers’ and those who answered ‘yes’ were further classified by a second question: ‘Have you smoked at least one cigarette in the past 30 days?’ Those answering ‘no’ were classified as ‘former smokers’ and those answering ‘yes’ were classified as ‘current smokers’.

### Categories of participants

The grouping criteria were based on the combined answers from baseline and second survey, and resulted in the following categories: current smokers, participants stated they were current smokers at both interviews; new quitters, those who stated they were smokers at baseline and had quit smoking before the second interview; continuing ex-smokers, who stated they were former smokers at both interviews; and never smokers, those who stated they were never smokers at both interviews.

### Case ascertainment

Incident cases of lung cancer occurring between the second interview and the end of the follow-up period were identified by linkage with the Singapore Cancer Registry using unique national identification numbers. A total of 517 incident lung cancer cases were diagnosed among cohort members between the second interview and 31 December 2007. The majority (440 or 85.1%) of these were based on histological diagnoses, 52 (10.1%) cases were diagnosed clinically, and these were confirmed by manual review of pathology reports by trained research staff. A total of 25 (4.8%) cases were identified through death records. The site of origin and histological subtypes were categorised according to ICD-O (International Classification of Diseases for Oncology) code. Included in this study are the following subtypes: adenocarcinoma, squamous cell carcinoma, small-cell lung cancer and primary lung cancer with other or unspecified histology.

### Data analysis

A total of 63 257 subjects were interviewed at baseline. On the second survey, ∼17% were not contactable because of death or physical disability, or could not be reached after three attempts by mail, phone call or house visit. A total of 52 324 subjects were successfully re-contacted, and the mean period between interviews was 5.8 years. Any subject with existing cancer diagnosed before second survey was excluded (*n*=2519). Participants who started smoking after baseline interview (*n*=1502) and those whose responses on both occasions were deemed inconsistent (*n*=2403) were also excluded, leaving a final study population of 45 900 subjects, among whom 463 incident cases of lung cancer were identified.

For each subject, person-years of follow-up were counted from the date of the second interview to the date of diagnosis of lung cancer, death or 31 December 2007, whichever occurred first. Proportional hazards (Cox) regression methods were used to examine the association between smoking cessation and risk of lung cancer. We adjusted simultaneously for age at interview (years), gender, dialect group (Hokkien, Cantonese), body mass index (BMI), year of recruitment, level of education (no formal education, primary school, secondary school or above) and daily intakes of ethanol (grams), fruits, vegetables and juices (grams), as well as daily dietary intakes of *β*-cryptoxanthin (*μ*g) and isothiocyanates (*μ*mol). The proportionality assumption was tested in the final model and found to be satisfied (*P*=0.45). Statistical computing was conducted using SAS version 9.1 (SAS Institute Inc., Cary, NC, USA) statistical software package. All *P*-values quoted were two sided, and *P*-values <0.05 were considered statistically significant.

## Results

Among 45 900 subjects in this analysis (18 688 men and 27 212 women), the prevalence rates of current and former smokers were 31.0 and 26.7% among men, and 4.2 and 2.4% for women, respectively. Ever smokers were older than never smokers. The results showed that current smokers and recent quitters had lower BMI at baseline. They were more likely to consume alcohol and reported lower consumption of vegetables and fruits compared with continuous ex-smokers and never smokers. Ever smokers were also less likely than never smokers to have no formal education (18.5 *vs* 28.2%) (Table 1). At baseline interview, continuous ex-smokers also reported the highest intensity of smoking in the past (32.6% reported >23 cigs/day) compared to current smokers (19.0%) and new quitters (15.9%) data not shown.

As of 31 December 2007, 463 cases of lung cancer were identified (314 male and 149 female). There were 177 adenocarcinomas (38.2%), 74 squamous cell carcinomas (16.0%), 47 small cell carcinomas (10.2%) and 165 cases (35.6%) which were either of other histological subtypes (24.4%) or of unknown histology (11.2%). Compared with current smokers (Table 2), individuals who quit smoking before the second survey had a 28% decrease in risk of lung cancer (adjusted hazard ratio (HR) 0.72; 95% confidence interval (95% CI): 0.53–0.98). Those who quit before baseline and maintained their status had less than half the risk of current smokers (HR 0.42; 95% CI: 0.32–0.56). The relative risk of lung cancer among never smokers was 0.14 (95% CI: 0.11–0.18). When the analysis was limited to smokers and further adjusted for smoking intensity and duration at baseline, the estimates were not materially changed (OR for new quitters 0.75, 95% CI 0.55–1.03; continuous ex-smokers 0.54, 95% CI 0.39–0.74). When stratified by gender, the reduction in risk remained strong and statistically significant among males. The numbers of female smokers and ex-smokers in this population were too small for a separate analysis to be meaningful.

## Discussion

From the perspective of cancer prevention, our results confirm that quitting smoking and maintaining that status is associated with a significant reduction in lung cancer risk in this Asian population. Importantly, this benefit can be observed within 6 years of quitting. Evidence from this, and other studies is therefore consistent with the understanding that although some of the genetic damage caused by smoking is irreversible and accounts for the higher risk that persists among ex-smokers, cessation of smoking reduces the period of time for which the bronchial epithelium is exposed to mutagens, and to the chronic inflammatory state which promotes carcinogenesis (Lee *et al*, 2009).

Although we did not attempt to verify smoking (and quitting) status by measuring cotinine levels or through other means, an important feature of the current study is the use of repeated measurements and the application of stringent criteria for classification of smoking status. Smoking behaviour, being dynamic, may change in an unpredictable manner, and reliance on one measurement time point may obscure true effects on disease risk.

The short-term (<5 years) benefits of smoking cessation are arguably more difficult to demonstrate because of the possibility that individuals may quit because of health problems that may pre-date a subsequent diagnosis of lung cancer. Earlier studies, both cohort and case–control, have been inconclusive with respect to short-term benefits, although the long-term reduction in risk is clear (International Agency for Research on Cancer, 2007). Such a bias may indeed be present in this study, and may have led to an underestimation of the true effect.

Among the limitations of this analysis are that the information collected as baseline was categorical in nature, and that the actual changes in the intensity of smoking could not be computed in a quantitative manner. The relatively short period for ascertainment of incident cases precluded a more detailed analysis by gender, or by histological subtype, both of which could yield important and relevant information.

Although the prevalence of smoking is decreasing in developed countries, there are worrying observations that in developing countries such as China (Yang, 1997; Liu *et al*, 1998) and India (Reddy *et al*, 2006), individuals are smoking higher doses and also starting at an earlier age. Hence, the estimated lung cancer mortality attributed to smoking in developing Asian countries can be expected to increase rapidly. Our data confirm that the beneficial effect of smoking cessation in Asian populations is likely to be substantial and can be demonstrated within a relatively short time after quitting. These findings are relevant for Asian populations at a similar stage in the tobacco epidemic curve, and underscore the importance of public health measures not only to prevent initiation of smoking but also to find effective means of helping current smokers to quit.

## Figures and Tables

**Table 1:** Baseline characteristics of 45 900 participants (mean and s.d. or number and %) by smoking category in the Singapore Chinese Health Study (1993–2007)

		**Smoking category**			
**Demographics at baseline recruitment**	**Total (*n*=45 900)**	**Current smokers (*n*=6955)**	**New quitters (*n*=1765)**	**Continuous ex-smokers (*n*=3888)**	**Never smokers (*n*=33 292)**
Men	18 688 (40.7%)	5802 (83.4%)	1495 (84.7%)	3503 (90.1%)	7888 (23.7%)
Age (years)	55.5 (7.7)	56.2 (7.5)	57.7 (7.6)	59.0 (7.9)	54.9 (7.5)
Body mass index	23.1 (3.3)	22.4 (3.2)	22.9 (3.3)	23.5 (3.4)	23.3 (3.3)
Dialect group, Cantonese	22 219 (48.4%)	2714 (39.0%)	719 (40.7%)	1988 (51.1%)	16798 (50.5%)
No formal education	11 731 (25.6%)	1467 (21.1%)	340 (19.3%)	523 (13.5%)	9401 (28.2%)
Alcohol intake, g per day	1.6 (7.3)	5.2 (13.5)	3.1 (10.3)	3.0 (10.5)	0.7 (3.6)
Vegetable intake, g per day	112.9 (63.3)	104.4 (61.5)	105.7 (61.7)	114.6 (65.7)	114.9 (63.2)
Fruit intake, g per day	207.0 (168.6)	170.7 (159.7)	186.1 (155.0)	219.8 (172.4)	214.2 (196.6)
Mean time between two interviews, years	5.8 (1.5)	6.0 (1.5)	6.3 (1.5)	5.6 (1.5)	5.7 (1.5)

**Table 2:** Lung cancer risk by smoking category among 45 900 participants in the Singapore Chinese Health Study (1993–2007)

		**Smoking category**			
	**Total**	**Current smokers**	**New quitters**	**Continuous ex-smokers**	**Never smokers**
No. at risk (%)	45 900	6955 (15.1%)	1765 (3.9%)	3888 (8.5%)	33 292 (72.5%)
Person-years of follow-up	290 832	41 798	10 588	23 573	214 873
Follow-up years, mean (s.d.)	6.3 (1.5)	6.0 (1.7)	6.0 (1.8)	6.1 (1.6)	6.5 (1.4)
					
*Lung cancer risk*					
*All cases*					
No. of cases (% of total cases)	463	234 (50.5%)	48 (10.4%)	63 (13.6%)	118 (25.5%)
Crude hazard ratio (95% CI)		1.0 (reference)	0.81 (0.59–1.10)	0.48 (0.36–0.63)	0.10 (0.08–0.12)
Adjusted hazard ratio^a^ (95% CI)		1.0 (reference)	0.72 (0.53–0.98)	0.42 (0.32–0.56)	0.14 (0.11–0.18)
*Male only*					
No. of cases (% in each smoking category)	314	199 (63.4%)	38 (12.1%)	53 (16.9%)	24 (7.6%)
Crude hazard ratio (95% CI)		1.0 (reference)	0.74 (0.52–1.05)	0.44 (0.32–0.59)	0.08 (0.06–0.13)
Adjusted hazard ratio^a^ (95% CI)		1.0 (reference)	0.64 (0.45–0.91)	0.38 (0.28–0.52)	0.11 (0.07–0.17)

Abbreviations: BMI=body mass index; CI=confidence interval.

a Multivariate model adjusted for gender (male, female), dialect group (Cantonese, Hokkien), age at recruitment (continuous), year at interview (continuous), BMI at baseline (continuous), education (no formal education, primary, secondary and above), ethanol intake (continuous), vegetable intake (continuous), fruit intake (continuous), dietary intake of *β*-cryptoxanthin (continuous) and isothiocyanates (continuous).
